# 4-Amino-5-(4-bromo­benzo­yl)-3-(benzo[*d*]thia­zol-2-yl)-2-[(2′,3′,4′,6′-tetra-*O*-acetyl-β-d-galacto­pyran­osyl­)sulfanyl]­thio­phene

**DOI:** 10.1107/S2414314622004126

**Published:** 2022-04-28

**Authors:** Rasha A. Azzam, Galal H. Elgemeie, Nagwa M. Gad, Peter G. Jones

**Affiliations:** a Chemistry Department, Faculty of Science, Helwan University, Cairo, Egypt; b Institut für Anorganische und Analytische Chemie, Technische Universität Braunschweig, Hagenring 30, D-38106 Braunschweig, Germany; Tulane University, USA

**Keywords:** benzo­thia­zole, thio­phene, galactose, crystal structure

## Abstract

In the title compound, C_32_H_29_BrN_2_O_10_S_3_, the benzo­thia­zole and thio­phene ring systems subtend an inter­planar angle of 7.43 (12)°. The NH_2_ group forms intra­molecular hydrogen bonds to N_thia­zole_ and O_carbon­yl_. The S_galactose_—C_thio­phene_ bond is short [1.759 (2) Å]. The mol­ecules are connected to form ribbons parallel to the *b* axis by two ‘weak’ hydrogen bonds and a short N_amino_⋯S_galactose_ contact.

## Abstract 2

The benzo­thia­zole and thio­phene rings are almost coplanar. The NH_2_ group forms intra­molecular hydrogen bonds. The S_galactose_—C_thio­phene_ bond is short. The mol­ecules are connected by two ‘weak’ hydrogen bonds and a short N⋯S contact.

## Graphical abstract

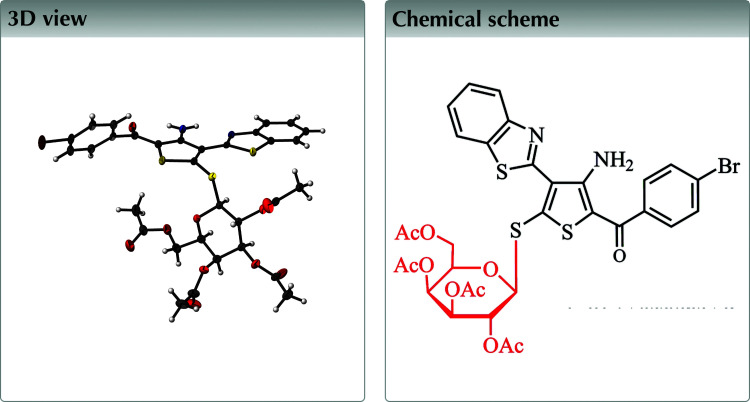

## Structure description

Benzo­thia­zoles are the most widely applied class of heterocyclic compounds in medicinal chemistry, and benzo­thia­zole derivatives have been employed in many pharmaceutical preparations (Bonde *et al.*, 2015 ▸), because they offer a variety of pharmacological properties useful in treating many diseases (Wang *et al.*, 2009 ▸). As clinical drugs, they often act with high therapeutic efficacy (Huang *et al.*, 2009 ▸). The broad pharmacological activities of benzo­thia­zoles suggest that they are also important for developing future drugs (Rana *et al.*, 2008 ▸). Recently we have explored various novel synthetic methods to obtain benzo­thia­zole derivatives (Azzam *et al.* 2017*a* ▸,*b* ▸, 2020*a* ▸,*b* ▸,*c* ▸, 2021 ▸; Elgemeie *et al.*, 2000*a* ▸,*b* ▸; 2020*a* ▸).

**Figure 1:**
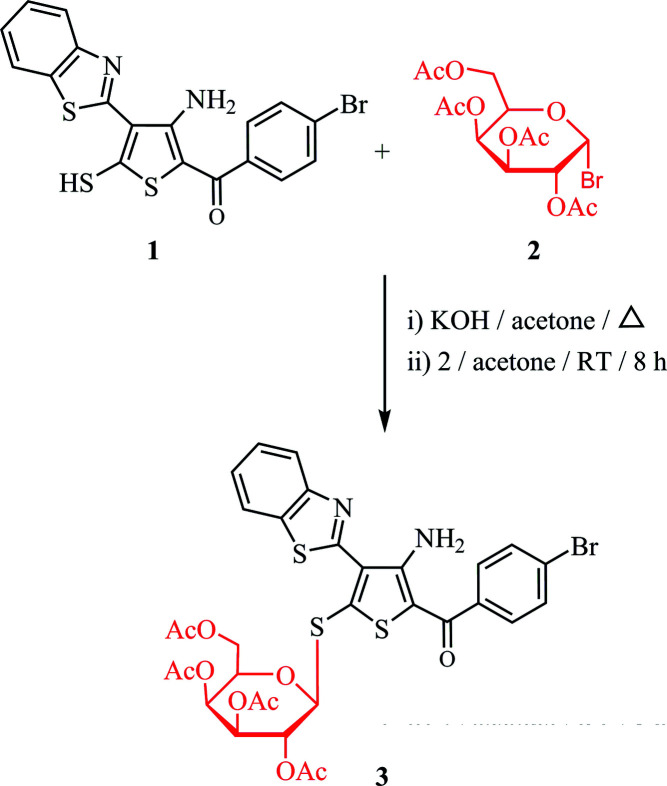
Reaction scheme.

As a part of our current plan directed toward discovering synthetic methodologies for the preparation of *S*-glycosyl­ated derivatives of heterocyclic nitro­gen bases (Elgemeie *et al.*, 2017*a* ▸,*b* ▸,*c* ▸), we have lately described the synthesis and biological activity of a series of heterocyclic *S*-glycosides that have promising cytotoxic activity (Abu-Zaied *et al.*, 2011 ▸, 2019*a* ▸,*b* ▸, 2020 ▸, 2021 ▸; Elgemeie *et al.*, 2009 ▸, 2018 ▸). We found that our reported di­hydro­pyridine *S*-glycosides have a strong anti-*P*-glycoprotein effect against human tumor cells (Scala *et al.*, 1997 ▸). Consistent with these outcomes and our past research (Elgemeie *et al.*, 2015 ▸, 2016 ▸, 2019 ▸, 2020*b* ▸), the purpose of the current study was to design and synthesize benzo­thia­zole-based thio­phene thio­glycosides. The synthesis of our target benzo­thia­zole-2-thio­phene thio­glycoside was carried out by the reaction of benzo­thia­zole 2-thio­phene­thiol derivative **1** with 2,3,4,6-tetra-*O*-acetyl-*β*-d-galacto­pyranosyl bromide **2** in the presence of potassium hydroxide to give the corresponding benzo­thia­zole-2-thio­phene *S*-glycoside **3** in good yield (Fig. 1 ▸). It has been suggested that the *cis*-(α) sugars react *via* a simple S_N_2 reaction to give the β-glycoside products such as **3** (Masoud *et al.*, 2017 ▸; Hammad *et al.*, 2018 ▸). The structure of **3** was confirmed based on the spectroscopic data (^13^C NMR, ^1^H NMR, and IR). The ^1^H NMR spectrum of compound **3** showed the anomeric proton as a doublet at δ = 5.39 p.p.m. with a spin–spin coupling constant (*J*_1′,2′_ = 8.8 Hz) confirming the β-configuration. The other six protons of galactose resonated at δ 4.00–5.30 p.p.m. In order to establish the structure of the product unambiguously, its crystal structure was determined and is reported here. To the best of our knowledge, this is the first reported X-ray structure of the new compound type benzo­thia­zole-2-thio­phene thio­glycoside.

**Figure 2:**
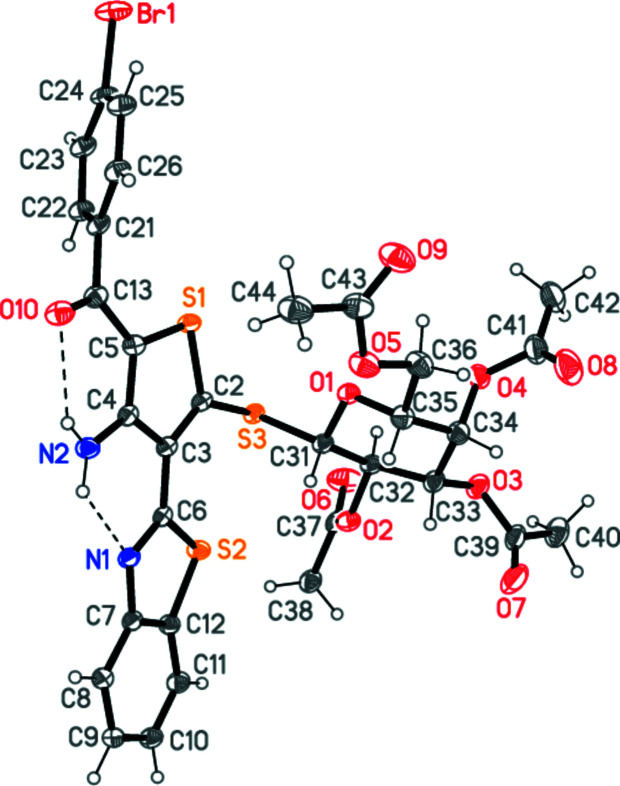
The mol­ecule of **3** in the crystal. Ellipsoids represent 50% probability levels. The dashed lines indicate intra­molecular hydrogen bonds.

The structure of **3** is shown in Fig. 2 ▸. The dimensions of the benzo­thia­zole moiety are as expected (a selection of mol­ecular dimensions is presented in Table 1 ▸). The benzo­thia­zole and thio­phene ring systems are approximately coplanar [inter­planar angle 7.43 (12)°], a geometry that is reinforced by the two intra­molecular hydrogen bonds from the NH_2_ group to the thia­zole nitro­gen atom and the C=O group (Table 2 ▸), whereas the bromo­phenyl and thio­phene rings subtend an angle of 58.23 (6)°. The intra­molecular S2⋯S3 contact is 3.1416 (8) Å.

The β configuration (equatorial position of the sulfur atom) at the anomeric carbon of the sugar (here C31) is confirmed, as is the axial configuration of the substituent at C34, characteristic of galactose. The galactose ring displays a slightly flattened chair conformation, with absolute torsion angles < 50° about C32—C33 and C33—C34. The configurations at C31–C35 are *S*, *R*, *S*, *R*, *R*, respectively. The S3—C31 bond is as expected longer than S3—C2, with values of 1.819 (2) and 1.759 (2) Å, respectively; the latter is significantly shorter than the values found for similar compounds in search of the Cambridge Structural Database (Groom *et al.*, 2016 ▸; performed using *CONQUEST* Version 2021.3.0) for purely organic galactose derivatives substituted with a sulfur atom at the anomeric carbon. There were 22 hits, of which two were axially substituted (NODQEC, Khiar *et al.*, 1997 ▸; YINFUY, Smith *et al.*, 2013 ▸) and the remainder equatorially substituted. The 29 C—S bond lengths for the latter lay in the range 1.788–1.856, average 1.808 (13) Å. Restricting the analysis to the ten hits with an *sp* ^2^ carbon atom altered these values only marginally.

**Figure 3:**
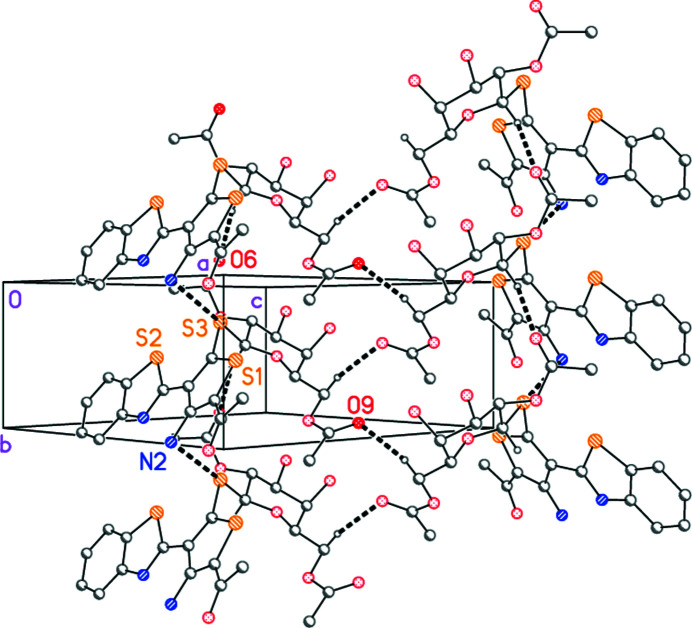
Crystal packing of **3** viewed perpendicular to (10 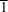 ) in the region *x* ≃ 0.75, *z* ≃ 0.25. For clarity, the following atoms/groups have been omitted: Hydrogen atoms not involved in ‘weak’ hydrogen bonding; two acetyl groups; the bromo­phenyl groups (except the *ipso* carbon atom). Dashed lines indicate hydrogen bonds or N⋯S contacts.

The N—H donor groups do not participate in inter­molecular hydrogen bonding, but two short and acceptably linear C—H⋯O contacts between the galactose moieties may be classed as ‘weak’ hydrogen bonds (Table 2 ▸). Additionally, a short contact N2⋯S3 of 3.249 (2) Å is observed (operator *x*, 1 + *y*, *z*). The net effect is to form ribbons of mol­ecules parallel to the *b* axis (Fig. 3 ▸).

## Synthesis and crystallization

Thio­phene thiol derivative **1** (2.23 g, 5 mmol) was dissolved in acetone (10 ml) containing 0.5 ml of aq. KOH (0.25 g, 5 mmol). The mixture was warmed to 50°C for 15 min. After cooling, a solution of 2,3,4,6-tetra-*O*-acetyl-*β*-d-galacto­pyranosyl bromide **2** (2.05 g, 5 mmol) in acetone (10 ml) was added dropwise over 30 min. The reaction mixture was stirred at room temperature and monitored by TLC until the reaction was complete (8 h). The residue was washed with distilled water to remove KBr, then dried and crystallized from ethanol to produce compound **3** (Fig. 1 ▸).

Yellow solid, yield 65%, m.p. 403–405 K (EtOH); IR (KBr, cm^−1^): ν 3406–3281 (NH_2_), 2923 (ArCH), 1748 (4Ac-CO), 1720 (CO); ^1^H NMR (400 MHz, DMSO-*d*_6_): δ 1.89, 1.91, 1.94, 2.01 (4 *s*, 12H, 4 × OAc), 4.00–4.02 (*m*, 2H, H-6′), 4.32 (*t*, *J* = 6.0 Hz, 1H, H-5′), 5.15 (*t*, *J* = 8.0 Hz, 1H, H-4′), 5.25–5.30 (*m*, 2H, H-3′, H-2′), 5.39 (*d*, *J* = 8.8 Hz, 1H, H-1′), 7.52 (*t*, *J* = 7.4 Hz, 1H, benzo­thia­zole-H), 7.61 (*t*, *J* = 7.4 Hz, 1H, benzo­thia­zole-H), 7.77–7.79 (*m*, 4H, Ar—H), 8.14 (*d*, *J* = 7.6 Hz, 1H, benzo­thia­zole-H), 8.21 (*d*, *J* = 8.0 Hz, 1H, benzo­thia­zole-H), 8.93 (*s*, *br*, D_2_O exch., 2H, NH_2_); Analysis: calculated for C_32_H_29_ BrN_2_O_10_S_3_ (777.68): C, 49.42; H, 3.76; N, 3.60; S, 12.37%. Found: C, 49.39; H, 3.73; N, 3.67; S, 12.40%.

## Refinement

Crystal data, data collection and structure refinement details are summarized in Table 3 ▸.

## Supplementary Material

Crystal structure: contains datablock(s) I, global. DOI: 10.1107/S2414314622004126/mw2186sup1.cif

Structure factors: contains datablock(s) I. DOI: 10.1107/S2414314622004126/mw2186Isup2.hkl

CCDC reference: 2167334

Additional supporting information:  crystallographic information; 3D view; checkCIF report

## Figures and Tables

**Table 1:** Selected geometric parameters (Å, °)

S1—C2	1.703 (2)	S2—C12	1.733 (2)
S1—C5	1.731 (2)	S3—C2	1.759 (2)
S2—C6	1.762 (2)	S3—C31	1.819 (2)
			
C2—S1—C5	92.15 (12)	N1—C6—S2	115.18 (17)
C12—S2—C6	88.99 (12)	N1—C7—C12	114.8 (2)
C2—S3—C31	98.36 (11)	C7—C12—S2	109.94 (17)
C6—N1—C7	111.1 (2)		
			
O1—C31—C32—C33	53.1 (2)	C32—C33—C34—C35	48.1 (3)
C31—O1—C35—C34	69.4 (2)	C33—C34—C35—O1	−57.9 (3)
C31—C32—C33—C34	−45.9 (3)	C35—O1—C31—C32	−66.0 (2)

**Table 2:** Hydrogen-bond geometry (Å, °)

*D*—H⋯*A*	*D*—H	H⋯*A*	*D*⋯*A*	*D*—H⋯*A*
N2—H01⋯N1	0.86 (3)	2.12 (3)	2.746 (3)	129 (3)
N2—H02⋯O10	0.86 (3)	2.14 (3)	2.795 (3)	133 (3)
C31—H31⋯O6^i^	1.00	2.34	3.290 (3)	158
C36—H36*B*⋯O9^ii^	0.99	2.33	3.294 (4)	164

Symmetry codes: (i) 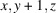 ; (ii) 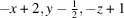 .

**Table 3:** Experimental details

Crystal data	
Chemical formula	C_32_H_29_BrN_2_O_10_S_3_
*M*_r_	777.66
Crystal system, space group	Monoclinic, *P*2_1_
Temperature (K)	100
*a*, *b*, *c* (Å)	16.99261 (18), 6.02635 (7), 17.4076 (2)
β (°)	107.8307 (12)
*V* (Å^3^)	1696.97 (3)
*Z*	2
Radiation type	Cu *K*α
μ (mm^−1^)	3.89
Crystal size (mm)	0.15 × 0.06 × 0.02

Data collection	
Diffractometer	XtaLAB Synergy
Absorption correction	Multi-scan (*CrysAlis PRO*; Rigaku OD, 2021 ▸)
*T*_min_, *T*_max_	0.781, 1.000
No. of measured, independent and observed [*I* > 2σ(*I*)] reflections	107906, 7129, 7052
*R*_int_	0.032
(sin θ/λ)_max_ (Å^−1^)	0.634

Refinement	
*R*[*F* ^2^ > 2σ(*F* ^2^)], *wR*(*F* ^2^), *S*	0.023, 0.062, 1.04
No. of reflections	7129
No. of parameters	445
No. of restraints	2
H-atom treatment	H atoms treated by a mixture of independent and constrained refinement
Δρ_max_, Δρ_min_ (e Å^−3^)	0.37, −0.72
Absolute structure	Flack *x* determined using 3095 quotients [(*I* ^+^)−(*I* ^−^)]/[(*I* ^+^)+(*I* ^−^)] (Parsons et al., 2013 ▸)
Absolute structure parameter	−0.019 (7)

Computer programs: *CrysAlis PRO* (Rigaku OD, 2021 ▸), *SHELXT* (Sheldrick, 2015*a* ▸), *SHELXL2018/3* (Sheldrick, 2015*b* ▸), *XP* (Siemens, 1994 ▸) and *OLEX2* (Dolomanov *et al.*, 2009 ▸).

## full crystallographic data

### Crystal data

**Table:**

C_32_H_29_BrN_2_O_10_S_3_	*F*(000) = 796
*M**_r_* = 777.66	*D*_x_ = 1.522 Mg m^−^^3^
Monoclinic, *P*2_1_	Cu *K*α radiation, λ = 1.54184 Å
*a* = 16.99261 (18) Å	Cell parameters from 91570 reflections
*b* = 6.02635 (7) Å	θ = 2.7–77.5°
*c* = 17.4076 (2) Å	µ = 3.89 mm^−^^1^
β = 107.8307 (12)°	*T* = 100 K
*V* = 1696.97 (3) Å^3^	Lath, pale yellow
*Z* = 2	0.15 × 0.06 × 0.02 mm

### Data collection

**Table:**

XtaLAB Synergy diffractometer	7129 independent reflections
Radiation source: micro-focus sealed X-ray tube, PhotonJet (Cu) X-ray Source	7052 reflections with *I* > 2σ(*I*)
Mirror monochromator	*R*_int_ = 0.032
Detector resolution: 10.0000 pixels mm^-1^	θ_max_ = 77.8°, θ_min_ = 2.7°
ω scans	*h* = −21→21
Absorption correction: multi-scan (CrysAlisPro; Rigaku OD, 2021)	*k* = −7→7
*T*_min_ = 0.781, *T*_max_ = 1.000	*l* = −22→22
107906 measured reflections	

### Refinement

**Table:**

Refinement on *F*^2^	Hydrogen site location: mixed
Least-squares matrix: full	H atoms treated by a mixture of independent and constrained refinement
*R*[*F*^2^ > 2σ(*F*^2^)] = 0.023	*w* = 1/[σ^2^(*F*_o_^2^) + (0.0397*P*)^2^ + 0.6757*P*] where *P* = (*F*_o_^2^ + 2*F*_c_^2^)/3
*wR*(*F*^2^) = 0.062	(Δ/σ)_max_ = 0.003
*S* = 1.04	Δρ_max_ = 0.37 e Å^−^^3^
7129 reflections	Δρ_min_ = −0.72 e Å^−^^3^
445 parameters	Absolute structure: Flack *x* determined using 3095 quotients [(*I*^+^)-(*I*^-^)]/[(*I*^+^)+(*I*^-^)] (Parsons et al., 2013)
2 restraints	Absolute structure parameter: −0.019 (7)
Primary atom site location: dual	

### Special details

**Table:**

Geometry. All esds (except the esd in the dihedral angle between two l.s. planes) are estimated using the full covariance matrix. The cell esds are taken into account individually in the estimation of esds in distances, angles and torsion angles; correlations between esds in cell parameters are only used when they are defined by crystal symmetry. An approximate (isotropic) treatment of cell esds is used for estimating esds involving l.s. planes. Short contacts: 3.1416 (0.0008) S2 - S3 3.2493 (0.0021) N2 - S3_$2 Operator $2 : x,1+y,z ============================================================================== Least-squares planes (x,y,z in crystal coordinates) and deviations from them (* indicates atom used to define plane) - 1.7949 (0.0074) x + 2.7373 (0.0043) y + 15.2223 (0.0070) z = 2.9267 (0.0054) * 0.0402 (0.0013) C2 * -0.0135 (0.0017) N1 * -0.0086 (0.0019) C6 * -0.0308 (0.0020) C7 * -0.0016 (0.0018) C8 * 0.0379 (0.0020) C9 * 0.0338 (0.0019) C10 * -0.0142 (0.0018) C11 * -0.0432 (0.0020) C12 Rms deviation of fitted atoms = 0.0288 - 0.0073 (0.0162) x + 3.1508 (0.0048) y + 14.1284 (0.0110) z = 4.2446 (0.0123) Angle to previous plane (with approximate esd) = 7.434 ( 0.118 ) * 0.0061 (0.0010) S1 * 0.0046 (0.0013) C2 * -0.0152 (0.0014) C3 * 0.0207 (0.0014) C4 * -0.0162 (0.0013) C5 0.0201 (0.0033) S3 0.0387 (0.0033) N2 -0.1146 (0.0038) C13 0.0343 (0.0044) O10 Rms deviation of fitted atoms = 0.0140 5.5692 (0.0163) x + 2.3719 (0.0054) y - 15.9799 (0.0068) z = 5.2155 (0.0164) Angle to previous plane (with approximate esd) = 58.230 ( 0.061 ) * -0.0124 (0.0016) C21 * -0.0049 (0.0017) C22 * 0.0186 (0.0017) C23 * -0.0152 (0.0018) C24 * -0.0023 (0.0018) C25 * 0.0162 (0.0017) C26 -0.0309 (0.0037) C13 0.8520 (0.0040) O10 -0.1298 (0.0035) Br1 Rms deviation of fitted atoms = 0.0130
Refinement. The hydrogen atoms of the NH_2_ group were refined freely, but with N—H distances restrained to be approximately equal (SADI). The methyl groups were refined as idealized rigid groups allowed to rotate but not tip, with C—H 0.98 Å and H—C—H 109.5 °. Other hydrogens were included using a riding model starting from calculated positions (C—H_aromatic_ 0.95, C—H_methylene_ 0.99, C—H_methine_ 1.00 Å). The *U*(H) values were fixed at 1.5 or 1.2 times the equivalent *U*_iso_ value of the parent carbon atoms for methyl and non-methyl hydrogens respectively.

### Fractional atomic coordinates and isotropic or equivalent isotropic displacement parameters (Å^2^)

**Table:**

	*x*	*y*	*z*	*U*_iso_*/*U*_eq_	
Br1	1.17709 (2)	0.30260 (5)	0.13689 (2)	0.03408 (9)	
S1	0.81796 (3)	0.50666 (10)	0.18829 (3)	0.01870 (11)	
S2	0.51931 (3)	0.50194 (10)	0.15908 (3)	0.01913 (11)	
S3	0.69078 (3)	0.27394 (9)	0.24112 (3)	0.01767 (11)	
O1	0.76755 (10)	0.4911 (3)	0.37404 (9)	0.0193 (3)	
O2	0.56621 (10)	0.2394 (3)	0.34351 (10)	0.0210 (3)	
O3	0.64615 (12)	0.2015 (3)	0.51551 (11)	0.0263 (4)	
O4	0.80626 (10)	0.3125 (3)	0.53459 (9)	0.0236 (3)	
O5	0.85216 (11)	0.8993 (3)	0.41271 (12)	0.0287 (4)	
O6	0.57638 (13)	−0.1299 (4)	0.33101 (15)	0.0391 (5)	
O7	0.55812 (15)	0.4238 (4)	0.55315 (13)	0.0413 (5)	
O8	0.86193 (15)	0.5092 (5)	0.64867 (13)	0.0468 (6)	
O9	0.98997 (13)	0.9040 (4)	0.46564 (14)	0.0394 (5)	
O10	0.85254 (11)	1.0388 (3)	0.07162 (11)	0.0243 (4)	
N1	0.54299 (12)	0.8799 (3)	0.09717 (11)	0.0180 (4)	
N2	0.69030 (12)	1.0127 (4)	0.07769 (12)	0.0196 (4)	
H01	0.6390 (17)	1.048 (6)	0.0665 (18)	0.023 (8)*	
H02	0.726 (2)	1.093 (6)	0.065 (2)	0.039 (10)*	
C2	0.71837 (13)	0.4982 (4)	0.19001 (13)	0.0177 (4)	
C3	0.67073 (14)	0.6741 (4)	0.14936 (13)	0.0167 (4)	
C4	0.71933 (13)	0.8253 (4)	0.11822 (12)	0.0171 (4)	
C5	0.80082 (14)	0.7495 (4)	0.13255 (13)	0.0188 (5)	
C6	0.58211 (14)	0.7040 (4)	0.13374 (13)	0.0166 (4)	
C7	0.45902 (14)	0.8671 (4)	0.08844 (13)	0.0174 (4)	
C8	0.40068 (14)	1.0310 (4)	0.05402 (13)	0.0204 (5)	
H8	0.416201	1.161819	0.031863	0.025*	
C9	0.31929 (14)	0.9979 (5)	0.05296 (14)	0.0220 (5)	
H9	0.278981	1.108347	0.030320	0.026*	
C10	0.29589 (13)	0.8040 (5)	0.08480 (13)	0.0226 (5)	
H10	0.240008	0.785287	0.083586	0.027*	
C11	0.35290 (15)	0.6397 (4)	0.11791 (14)	0.0215 (5)	
H11	0.336876	0.507638	0.138925	0.026*	
C12	0.43461 (14)	0.6732 (4)	0.11962 (13)	0.0186 (4)	
C13	0.86263 (14)	0.8523 (4)	0.10269 (14)	0.0196 (5)	
C21	0.93984 (14)	0.7229 (4)	0.10924 (14)	0.0192 (5)	
C22	0.93411 (14)	0.5116 (4)	0.07541 (14)	0.0214 (5)	
H22	0.881300	0.450400	0.048610	0.026*	
C23	1.00516 (15)	0.3892 (4)	0.08054 (15)	0.0221 (5)	
H23	1.001565	0.247432	0.055855	0.026*	
C24	1.08128 (14)	0.4797 (5)	0.12261 (15)	0.0241 (5)	
C25	1.08860 (15)	0.6910 (5)	0.15573 (16)	0.0248 (5)	
H25	1.141475	0.750716	0.183250	0.030*	
C26	1.01739 (14)	0.8141 (5)	0.14802 (14)	0.0219 (5)	
H26	1.021491	0.960444	0.169162	0.026*	
C31	0.68674 (14)	0.4215 (4)	0.33099 (14)	0.0177 (4)	
H31	0.649650	0.553540	0.315148	0.021*	
C32	0.65357 (13)	0.2649 (4)	0.38322 (13)	0.0189 (5)	
H32	0.682566	0.118429	0.390455	0.023*	
C33	0.66127 (15)	0.3731 (4)	0.46429 (14)	0.0211 (5)	
H33	0.617097	0.488234	0.456146	0.025*	
C34	0.74480 (15)	0.4824 (4)	0.50439 (14)	0.0225 (5)	
H34	0.740969	0.577656	0.550143	0.027*	
C35	0.76617 (15)	0.6258 (4)	0.44166 (14)	0.0221 (5)	
H35	0.722698	0.742486	0.422673	0.026*	
C36	0.84913 (16)	0.7367 (5)	0.47261 (16)	0.0277 (5)	
H36A	0.855276	0.809402	0.525127	0.033*	
H36B	0.894073	0.626681	0.479826	0.033*	
C37	0.53570 (15)	0.0359 (4)	0.31928 (14)	0.0222 (5)	
C38	0.44510 (15)	0.0517 (5)	0.27487 (15)	0.0255 (5)	
H38A	0.417097	0.130629	0.308282	0.038*	
H38B	0.421897	−0.097856	0.263079	0.038*	
H38C	0.437085	0.132674	0.224223	0.038*	
C39	0.59276 (16)	0.2497 (5)	0.55765 (15)	0.0279 (6)	
C40	0.5876 (2)	0.0572 (6)	0.61001 (17)	0.0354 (7)	
H40A	0.578982	−0.079620	0.578079	0.053*	
H40B	0.541162	0.079141	0.631549	0.053*	
H40C	0.639093	0.045837	0.654741	0.053*	
C41	0.86038 (18)	0.3428 (5)	0.60992 (15)	0.0326 (6)	
C42	0.9152 (2)	0.1462 (6)	0.63644 (17)	0.0375 (7)	
H42A	0.883168	0.022697	0.648177	0.056*	
H42B	0.960362	0.183859	0.685145	0.056*	
H42C	0.938204	0.102746	0.593438	0.056*	
C43	0.92782 (16)	0.9685 (5)	0.41543 (17)	0.0283 (5)	
C44	0.92423 (18)	1.1323 (5)	0.3502 (2)	0.0352 (6)	
H44A	0.890426	1.072087	0.298245	0.053*	
H44B	0.980271	1.161330	0.348046	0.053*	
H44C	0.899639	1.270909	0.361338	0.053*	

### Atomic displacement parameters (Å^2^)

**Table:**

	*U*^11^	*U*^22^	*U*^33^	*U*^12^	*U*^13^	*U*^23^
Br1	0.01892 (12)	0.02848 (15)	0.05928 (18)	0.00424 (11)	0.01851 (11)	0.00576 (13)
S1	0.0151 (2)	0.0187 (3)	0.0240 (2)	0.0015 (2)	0.00866 (19)	0.0033 (2)
S2	0.0149 (2)	0.0184 (3)	0.0248 (2)	−0.0013 (2)	0.00720 (19)	0.0027 (2)
S3	0.0188 (2)	0.0162 (3)	0.0201 (2)	0.0004 (2)	0.00916 (18)	0.0012 (2)
O1	0.0162 (7)	0.0215 (9)	0.0208 (7)	−0.0012 (7)	0.0064 (6)	−0.0001 (7)
O2	0.0178 (8)	0.0197 (9)	0.0268 (8)	−0.0013 (6)	0.0088 (6)	−0.0008 (6)
O3	0.0289 (9)	0.0290 (10)	0.0255 (8)	−0.0005 (8)	0.0151 (7)	0.0046 (7)
O4	0.0246 (8)	0.0248 (9)	0.0191 (7)	0.0013 (8)	0.0035 (6)	−0.0013 (7)
O5	0.0223 (9)	0.0254 (10)	0.0370 (10)	−0.0040 (7)	0.0071 (7)	0.0052 (8)
O6	0.0286 (10)	0.0248 (11)	0.0613 (13)	0.0015 (8)	0.0100 (9)	−0.0130 (9)
O7	0.0529 (14)	0.0450 (14)	0.0372 (11)	0.0105 (11)	0.0305 (10)	0.0039 (9)
O8	0.0534 (14)	0.0464 (14)	0.0312 (10)	0.0093 (12)	−0.0011 (9)	−0.0112 (10)
O9	0.0244 (10)	0.0413 (12)	0.0470 (12)	−0.0057 (9)	0.0030 (9)	0.0085 (10)
O10	0.0228 (8)	0.0182 (9)	0.0351 (9)	−0.0009 (7)	0.0136 (7)	0.0034 (7)
N1	0.0163 (9)	0.0204 (10)	0.0189 (8)	0.0004 (7)	0.0079 (7)	−0.0008 (7)
N2	0.0170 (9)	0.0187 (10)	0.0245 (9)	0.0019 (8)	0.0088 (7)	0.0051 (8)
C2	0.0165 (10)	0.0193 (11)	0.0194 (9)	−0.0007 (9)	0.0086 (8)	−0.0017 (9)
C3	0.0164 (10)	0.0167 (11)	0.0181 (10)	−0.0011 (8)	0.0071 (8)	−0.0014 (8)
C4	0.0177 (10)	0.0183 (11)	0.0166 (9)	−0.0018 (9)	0.0071 (8)	−0.0014 (9)
C5	0.0172 (10)	0.0188 (12)	0.0209 (10)	0.0000 (8)	0.0068 (8)	−0.0005 (8)
C6	0.0179 (11)	0.0180 (11)	0.0157 (9)	−0.0009 (8)	0.0079 (8)	−0.0008 (8)
C7	0.0157 (10)	0.0207 (12)	0.0167 (9)	−0.0014 (8)	0.0061 (8)	−0.0022 (8)
C8	0.0187 (11)	0.0239 (13)	0.0195 (10)	0.0021 (9)	0.0071 (8)	0.0024 (9)
C9	0.0180 (11)	0.0281 (13)	0.0201 (10)	0.0054 (10)	0.0063 (8)	0.0005 (10)
C10	0.0158 (9)	0.0303 (13)	0.0226 (10)	−0.0015 (11)	0.0073 (8)	−0.0014 (11)
C11	0.0173 (11)	0.0246 (13)	0.0240 (11)	−0.0039 (9)	0.0083 (9)	0.0001 (9)
C12	0.0160 (11)	0.0207 (12)	0.0191 (10)	−0.0002 (9)	0.0053 (8)	−0.0010 (9)
C13	0.0187 (10)	0.0193 (12)	0.0219 (10)	−0.0029 (8)	0.0076 (8)	−0.0023 (8)
C21	0.0190 (11)	0.0195 (11)	0.0227 (10)	−0.0026 (9)	0.0116 (9)	0.0016 (9)
C22	0.0182 (11)	0.0215 (12)	0.0267 (11)	−0.0021 (10)	0.0099 (9)	0.0004 (10)
C23	0.0211 (11)	0.0192 (11)	0.0299 (12)	−0.0015 (9)	0.0136 (9)	0.0003 (9)
C24	0.0159 (11)	0.0260 (13)	0.0346 (12)	0.0032 (10)	0.0138 (9)	0.0053 (11)
C25	0.0172 (11)	0.0260 (13)	0.0326 (13)	−0.0031 (10)	0.0097 (9)	−0.0001 (10)
C26	0.0210 (10)	0.0201 (12)	0.0266 (10)	−0.0026 (10)	0.0102 (9)	0.0007 (10)
C31	0.0165 (10)	0.0179 (11)	0.0200 (10)	−0.0003 (9)	0.0077 (8)	0.0010 (8)
C32	0.0162 (10)	0.0197 (13)	0.0223 (10)	−0.0010 (9)	0.0079 (8)	0.0010 (9)
C33	0.0224 (11)	0.0221 (12)	0.0215 (10)	−0.0003 (9)	0.0104 (9)	0.0028 (9)
C34	0.0242 (12)	0.0227 (12)	0.0210 (10)	0.0008 (10)	0.0075 (9)	−0.0019 (10)
C35	0.0222 (12)	0.0207 (12)	0.0228 (11)	−0.0014 (9)	0.0061 (9)	−0.0022 (9)
C36	0.0234 (12)	0.0269 (14)	0.0296 (12)	−0.0057 (10)	0.0035 (10)	0.0014 (10)
C37	0.0235 (12)	0.0234 (13)	0.0235 (11)	−0.0033 (10)	0.0128 (9)	−0.0038 (10)
C38	0.0216 (11)	0.0326 (15)	0.0242 (11)	−0.0063 (10)	0.0096 (9)	−0.0028 (10)
C39	0.0280 (12)	0.0387 (17)	0.0202 (11)	−0.0052 (11)	0.0122 (9)	−0.0025 (10)
C40	0.0403 (16)	0.0407 (18)	0.0300 (13)	−0.0078 (13)	0.0180 (12)	0.0024 (12)
C41	0.0381 (14)	0.0354 (17)	0.0215 (11)	0.0039 (12)	0.0050 (10)	−0.0017 (11)
C42	0.0414 (16)	0.0391 (17)	0.0253 (13)	0.0087 (13)	0.0002 (12)	−0.0012 (12)
C43	0.0227 (12)	0.0249 (13)	0.0361 (13)	−0.0042 (10)	0.0072 (10)	−0.0019 (11)
C44	0.0258 (13)	0.0321 (16)	0.0474 (16)	−0.0016 (12)	0.0109 (12)	0.0085 (13)

### Geometric parameters (Å, º)

**Table:**

Br1—C24	1.898 (2)	C24—C25	1.388 (4)
S1—C2	1.703 (2)	C25—C26	1.390 (4)
S1—C5	1.731 (2)	C31—C32	1.532 (3)
S2—C6	1.762 (2)	C32—C33	1.523 (3)
S2—C12	1.733 (2)	C33—C34	1.526 (3)
S3—C2	1.759 (2)	C34—C35	1.521 (3)
S3—C31	1.819 (2)	C35—C36	1.503 (3)
O1—C31	1.412 (3)	C37—C38	1.499 (3)
O1—C35	1.436 (3)	C39—C40	1.494 (4)
O2—C32	1.441 (3)	C41—C42	1.490 (4)
O2—C37	1.348 (3)	C43—C44	1.492 (4)
O3—C33	1.439 (3)	N2—H01	0.86 (3)
O3—C39	1.361 (3)	N2—H02	0.86 (3)
O4—C34	1.442 (3)	C8—H8	0.9500
O4—C41	1.364 (3)	C9—H9	0.9500
O5—C36	1.443 (3)	C10—H10	0.9500
O5—C43	1.338 (3)	C11—H11	0.9500
O6—C37	1.196 (3)	C22—H22	0.9500
O7—C39	1.194 (4)	C23—H23	0.9500
O8—C41	1.205 (4)	C25—H25	0.9500
O9—C43	1.210 (3)	C26—H26	0.9500
O10—C13	1.237 (3)	C31—H31	1.0000
N1—C6	1.308 (3)	C32—H32	1.0000
N1—C7	1.390 (3)	C33—H33	1.0000
N2—C4	1.343 (3)	C34—H34	1.0000
C2—C3	1.389 (3)	C35—H35	1.0000
C3—C4	1.442 (3)	C36—H36A	0.9900
C3—C6	1.457 (3)	C36—H36B	0.9900
C4—C5	1.406 (3)	C38—H38A	0.9800
C5—C13	1.446 (3)	C38—H38B	0.9800
C7—C8	1.398 (3)	C38—H38C	0.9800
C7—C12	1.404 (3)	C40—H40A	0.9800
C8—C9	1.392 (3)	C40—H40B	0.9800
C9—C10	1.402 (4)	C40—H40C	0.9800
C10—C11	1.382 (4)	C42—H42A	0.9800
C11—C12	1.394 (3)	C42—H42B	0.9800
C13—C21	1.500 (3)	C42—H42C	0.9800
C21—C22	1.394 (4)	C44—H44A	0.9800
C21—C26	1.396 (3)	C44—H44B	0.9800
C22—C23	1.394 (3)	C44—H44C	0.9800
C23—C24	1.388 (3)		
			
C2—S1—C5	92.15 (12)	O4—C41—C42	111.4 (2)
C12—S2—C6	88.99 (12)	O8—C41—O4	122.8 (3)
C2—S3—C31	98.36 (11)	O8—C41—C42	125.7 (3)
C31—O1—C35	110.22 (17)	O5—C43—C44	111.4 (2)
C37—O2—C32	119.22 (19)	O9—C43—O5	122.8 (3)
C39—O3—C33	117.1 (2)	O9—C43—C44	125.8 (3)
C41—O4—C34	117.1 (2)	C4—N2—H01	121 (2)
C43—O5—C36	115.6 (2)	C4—N2—H02	116 (3)
C6—N1—C7	111.1 (2)	H01—N2—H02	123 (3)
S1—C2—S3	116.88 (14)	C9—C8—H8	120.8
C3—C2—S1	113.32 (18)	C7—C8—H8	120.8
C3—C2—S3	129.80 (17)	C8—C9—H9	119.5
C2—C3—C4	111.2 (2)	C10—C9—H9	119.5
C2—C3—C6	126.7 (2)	C11—C10—H10	119.5
C4—C3—C6	122.0 (2)	C9—C10—H10	119.5
N2—C4—C3	124.6 (2)	C10—C11—H11	120.9
N2—C4—C5	123.3 (2)	C12—C11—H11	120.9
C5—C4—C3	112.0 (2)	C21—C22—H22	119.7
C4—C5—S1	111.20 (17)	C23—C22—H22	119.7
C4—C5—C13	125.6 (2)	C24—C23—H23	120.8
C13—C5—S1	123.20 (18)	C22—C23—H23	120.8
N1—C6—S2	115.18 (17)	C24—C25—H25	120.5
N1—C6—C3	122.2 (2)	C26—C25—H25	120.5
C3—C6—S2	122.57 (18)	C25—C26—H26	120.0
N1—C7—C8	125.2 (2)	C21—C26—H26	120.0
N1—C7—C12	114.8 (2)	O1—C31—H31	109.8
C8—C7—C12	120.0 (2)	C32—C31—H31	109.8
C9—C8—C7	118.4 (2)	S3—C31—H31	109.8
C8—C9—C10	121.0 (2)	O2—C32—H32	111.2
C11—C10—C9	121.0 (2)	C33—C32—H32	111.2
C10—C11—C12	118.2 (2)	C31—C32—H32	111.2
C7—C12—S2	109.94 (17)	O3—C33—H33	108.5
C11—C12—S2	128.6 (2)	C32—C33—H33	108.5
C11—C12—C7	121.5 (2)	C34—C33—H33	108.5
O10—C13—C5	121.9 (2)	O4—C34—H34	109.5
O10—C13—C21	120.4 (2)	C35—C34—H34	109.5
C5—C13—C21	117.7 (2)	C33—C34—H34	109.5
C22—C21—C13	119.8 (2)	O1—C35—H35	108.8
C22—C21—C26	119.8 (2)	C36—C35—H35	108.8
C26—C21—C13	120.4 (2)	C34—C35—H35	108.8
C21—C22—C23	120.6 (2)	O5—C36—H36A	110.5
C24—C23—C22	118.4 (2)	C35—C36—H36A	110.5
C23—C24—Br1	118.1 (2)	O5—C36—H36B	110.5
C23—C24—C25	122.0 (2)	C35—C36—H36B	110.5
C25—C24—Br1	119.82 (19)	H36A—C36—H36B	108.7
C24—C25—C26	119.0 (2)	C37—C38—H38A	109.5
C25—C26—C21	120.1 (2)	C37—C38—H38B	109.5
O1—C31—S3	108.36 (15)	H38A—C38—H38B	109.5
O1—C31—C32	110.05 (18)	C37—C38—H38C	109.5
C32—C31—S3	109.08 (16)	H38A—C38—H38C	109.5
O2—C32—C31	107.04 (18)	H38B—C38—H38C	109.5
O2—C32—C33	105.88 (18)	C39—C40—H40A	109.5
C33—C32—C31	110.08 (19)	C39—C40—H40B	109.5
O3—C33—C32	106.6 (2)	H40A—C40—H40B	109.5
O3—C33—C34	110.14 (19)	C39—C40—H40C	109.5
C32—C33—C34	114.36 (19)	H40A—C40—H40C	109.5
O4—C34—C33	109.2 (2)	H40B—C40—H40C	109.5
O4—C34—C35	111.1 (2)	C41—C42—H42A	109.5
C35—C34—C33	108.05 (19)	C41—C42—H42B	109.5
O1—C35—C34	109.6 (2)	H42A—C42—H42B	109.5
O1—C35—C36	107.2 (2)	C41—C42—H42C	109.5
C36—C35—C34	113.5 (2)	H42A—C42—H42C	109.5
O5—C36—C35	106.3 (2)	H42B—C42—H42C	109.5
O2—C37—C38	109.8 (2)	C43—C44—H44A	109.5
O6—C37—O2	124.0 (2)	C43—C44—H44B	109.5
O6—C37—C38	126.1 (2)	H44A—C44—H44B	109.5
O3—C39—C40	109.7 (2)	C43—C44—H44C	109.5
O7—C39—O3	123.3 (2)	H44A—C44—H44C	109.5
O7—C39—C40	127.0 (3)	H44B—C44—H44C	109.5
			
Br1—C24—C25—C26	−176.89 (19)	C7—N1—C6—S2	1.4 (2)
S1—C2—C3—C4	−2.1 (2)	C7—N1—C6—C3	179.0 (2)
S1—C2—C3—C6	174.13 (19)	C7—C8—C9—C10	−0.7 (3)
S1—C5—C13—O10	−171.04 (18)	C8—C7—C12—S2	−179.43 (18)
S1—C5—C13—C21	10.0 (3)	C8—C7—C12—C11	−0.6 (3)
S3—C2—C3—C4	178.18 (17)	C8—C9—C10—C11	−0.2 (4)
S3—C2—C3—C6	−5.6 (4)	C9—C10—C11—C12	0.7 (4)
S3—C31—C32—O2	−73.6 (2)	C10—C11—C12—S2	178.28 (19)
S3—C31—C32—C33	171.81 (16)	C10—C11—C12—C7	−0.3 (4)
O1—C31—C32—O2	167.69 (18)	C12—S2—C6—N1	−1.70 (18)
O1—C31—C32—C33	53.1 (2)	C12—S2—C6—C3	−179.36 (19)
O1—C35—C36—O5	70.6 (3)	C12—C7—C8—C9	1.1 (3)
O2—C32—C33—O3	76.8 (2)	C13—C21—C22—C23	179.7 (2)
O2—C32—C33—C34	−161.3 (2)	C13—C21—C26—C25	178.3 (2)
O3—C33—C34—O4	47.2 (2)	C21—C22—C23—C24	2.3 (4)
O3—C33—C34—C35	168.1 (2)	C22—C21—C26—C25	−2.6 (4)
O4—C34—C35—O1	61.9 (2)	C22—C23—C24—Br1	174.97 (18)
O4—C34—C35—C36	−58.0 (3)	C22—C23—C24—C25	−3.3 (4)
O10—C13—C21—C22	−123.4 (3)	C23—C24—C25—C26	1.4 (4)
O10—C13—C21—C26	55.7 (3)	C24—C25—C26—C21	1.6 (4)
N1—C7—C8—C9	−177.1 (2)	C26—C21—C22—C23	0.6 (3)
N1—C7—C12—S2	−1.1 (2)	C31—S3—C2—S1	105.40 (14)
N1—C7—C12—C11	177.7 (2)	C31—S3—C2—C3	−74.9 (2)
N2—C4—C5—S1	178.69 (18)	C31—O1—C35—C34	69.4 (2)
N2—C4—C5—C13	−3.7 (4)	C31—O1—C35—C36	−166.9 (2)
C2—S1—C5—C4	1.97 (18)	C31—C32—C33—O3	−167.85 (18)
C2—S1—C5—C13	−175.7 (2)	C31—C32—C33—C34	−45.9 (3)
C2—S3—C31—O1	−66.64 (17)	C32—O2—C37—O6	2.7 (3)
C2—S3—C31—C32	173.56 (15)	C32—O2—C37—C38	−176.21 (19)
C2—C3—C4—N2	−178.6 (2)	C32—C33—C34—O4	−72.8 (2)
C2—C3—C4—C5	3.6 (3)	C32—C33—C34—C35	48.1 (3)
C2—C3—C6—S2	−4.9 (3)	C33—O3—C39—O7	0.9 (4)
C2—C3—C6—N1	177.6 (2)	C33—O3—C39—C40	−177.5 (2)
C3—C4—C5—S1	−3.5 (2)	C33—C34—C35—O1	−57.9 (3)
C3—C4—C5—C13	174.1 (2)	C33—C34—C35—C36	−177.7 (2)
C4—C3—C6—S2	170.96 (17)	C34—O4—C41—O8	−5.0 (4)
C4—C3—C6—N1	−6.5 (3)	C34—O4—C41—C42	174.8 (2)
C4—C5—C13—O10	11.6 (4)	C34—C35—C36—O5	−168.2 (2)
C4—C5—C13—C21	−167.4 (2)	C35—O1—C31—S3	174.79 (15)
C5—S1—C2—S3	179.85 (14)	C35—O1—C31—C32	−66.0 (2)
C5—S1—C2—C3	0.13 (18)	C36—O5—C43—O9	−1.6 (4)
C5—C13—C21—C22	55.6 (3)	C36—O5—C43—C44	178.8 (2)
C5—C13—C21—C26	−125.3 (2)	C37—O2—C32—C31	119.2 (2)
C6—S2—C12—C7	1.47 (17)	C37—O2—C32—C33	−123.3 (2)
C6—S2—C12—C11	−177.2 (2)	C39—O3—C33—C32	−132.7 (2)
C6—N1—C7—C8	178.1 (2)	C39—O3—C33—C34	102.8 (2)
C6—N1—C7—C12	−0.2 (3)	C41—O4—C34—C33	−136.2 (2)
C6—C3—C4—N2	4.9 (3)	C41—O4—C34—C35	104.8 (3)
C6—C3—C4—C5	−172.8 (2)	C43—O5—C36—C35	−160.4 (2)

### Hydrogen-bond geometry (Å, º)

**Table:**

*D*—H···*A*	*D*—H	H···*A*	*D*···*A*	*D*—H···*A*
N2—H01···N1	0.86 (3)	2.12 (3)	2.746 (3)	129 (3)
N2—H02···O10	0.86 (3)	2.14 (3)	2.795 (3)	133 (3)
C11—H11···Br1^i^	0.95	2.97	3.710 (2)	135
C31—H31···O6^ii^	1.00	2.34	3.290 (3)	158
C35—H35···O6^ii^	1.00	2.62	3.532 (3)	151
C36—H36*B*···O9^iii^	0.99	2.33	3.294 (4)	164

Symmetry codes: (i) *x*−1, *y*, *z*; (ii) *x*, *y*+1, *z*; (iii) −*x*+2, *y*−1/2, −*z*+1.
